# A Level-Set Based Representative Volume Element Generator and XFEM Simulations for Textile and 3D-Reinforced Composites

**DOI:** 10.3390/ma6125568

**Published:** 2013-11-28

**Authors:** Bernard Sonon, Thierry J. Massart

**Affiliations:** Building, Architecture and Town Planning Departement (BATir) CP 194/2, Université Libre de Bruxelles (ULB), Avenue F.D. Roosevelt 50, 1050 Brussels, Belgium; E-Mail: bsonon@gmail.com

**Keywords:** textile-reinforced composites, RVE generation, level sets, extended finite elements, computational homogenization

## Abstract

This contribution presents a new framework for the computational homogenization of the mechanical properties of textile reinforced composites. A critical point in such computational procedures is the definition and discretization of realistic representative volume elements (RVEs). A geometrically-based weave generator has been developed to produce realistic geometrical configurations of the reinforcing textile. This generator takes into account the contact conditions between the yarns in the reinforcement by means of an iterative scheme, accommodating the tension in the yarns in an implicit manner. The shape of the cross sections of the yarns can also be adapted as a function of the contact conditions using a level set-based post-processor. This allows a seamless transition towards an extended finite element (XFE) scheme, in which the obtained reinforcement geometry is subsequently exploited to derive the mechanical properties of the composite system using computational homogenization.

## 1. Introduction

Composite materials based on textile reinforcements are nowadays considered for various industrial applications as a result of their high stiffness and strength performances at low density and with low manufacturing costs. Next to the use of two-dimensional reinforcements in laminated composites, new weaving techniques have emerged, thereby enabling complex three-dimensional woven reinforcements [1].

Various authors have presented the main advantages of 3D reinforcements, such as lower sensitivity to delamination and crack propagation [2]. The use of 3D woven-reinforced materials is however accompanied by an additional difficulty stemming from the complex geometry of their microstructure. This reinforcement geometry leads to an increase of the multiaxiality of the stress state inside the microstructure, which in combination with high volume fractions of reinforcement may cause the presence of voids after injection of resins (matrix) with a high viscosity. Reinforcements with higher waviness also seem to be more sensitive to variations in architecture than others as reported in [3]. Finally, the influence of the manufacturing parameters, among which the tensioning of the yarns, on the response of 3D interlock composites, was mentioned in [4].

For these new material systems to be used optimally, their average mechanical properties must be properly identified by means of experiments and/or simulations. The composite research community has not achieved so far a definition of universal standards for composite systems testing. The simulation of such material systems is therefore an active field of research for gaining understanding of 3D composite systems.

An extensive review of the modelling approaches developed for 3D-woven composites can be found in [5]. The extraction of the average (stiffness and strength) properties of 3D-reinforced composites by analytical approaches becomes quickly complex [6], as the yarn undulations are difficult to take into account analytically. These undulations were only incorporated approximately, see [7] for in-plane behavior or [8] for situations including bending. This has led to a large number of contributions based on computational homogenization developments in the past few years with extensive use of finite element approaches, which furnish local stress and strain fields in addition to the average properties of the studied composite systems. It has triggered various authors to assess different aspects, such as the effect of reinforcement architecture and interfacial debonding [9], the influence of crimp on the stiffness properties of 3D-woven composites [10], or the strain rate sensitivity in carbon epoxy woven composites [11]. The response of 3D-reinforced composites under biaxial loading was investigated in [12] accounting for matrix plasticity and debonding effects, while the effect of variations in braid parameters on the progressive failure of a 2 × 2 braided composite laminate was studied in [13]. Other approaches were also developed as in [14] with the method of cells to circumvent heavy finite element computations, while avoiding the uniform stress/strain approximations used by analytical models. The flexural properties of 3D-reinforced composites are even more complex to derive experimentally, which motivates their extraction by means of homogenization principles as performed recently in [4,15,16,17].

In any computational (homogenization) approach based on Representative Volume Elements (RVEs), a crucial point resides in the availability of a realistic geometrical representation of the reinforcing textile [4,18,19]. A potential procedure to obtain such a RVE representation is to resort to image-based finite element approaches in which X-ray tomography is used to reconstruct finite element meshes corresponding to the microstructure [20]. This however requires the actual manufacturing of the composite before the CT extraction. Therefore, other approaches have been developed to generate realistic geometries and meshes using a voxel-based methodology followed by a smoothing procedure [21]. From a general point of view, an over-simplification of the yarn’s geometry (position and section shape) often leads to overlaps (interpenetrations) between yarns in the obtained reinforcement configuration, which in turn prevent its use in analyses, as noted by [22]. Many of the available tools introduce strong assumptions, such as the approximation that the yarn’s cross sections remain constant. Depending on the nature of the yarns and on the local boundary and contact conditions, such an assumption may however not be valid in reality as noted by [18,22]. This triggered the development of dedicated tools to determine the geometrical configuration of the reinforcement as a pre-processing step before mechanical simulations. Lomov *et al.* [23,24] developed such a pre-processor in which the geometry of the reinforcement (yarn trajectory and cross-section) is computed based on a principle of minimum energy, and in which overlaps are prevented using an intermediate finite element calculation [25]. Aminimum energy procedure was also invoked in [26] to derive the geometrical configuration of yarns within a textile reinforcement. For the specific case of a layer-to-layer angle interlock weave, Mahadik and Hallett [27] developed a procedure for determining the internalgeometry after compaction. An alternative approach to model the strand path geometry prior to mechanical simulations was recently proposed in [22]. It incorporates the possibility of a smooth variation of the size and shape of the (initially circular) cross-section of reinforcing yarns. This tool models the strand perimeters as inflatable tubes in an explicit finite element computation together with contact conditions. These tubes are inflated to reach a prescribed volume fraction of reinforcement, and contact conditions are subsequently replaced by a clearance to allow the insertion of small volume elements between the yarns and thereby ease the meshing of the obtained RVE. This concept was applied in [22] for the homogenization of the RVE of the interior of a 3D-reinforced composite.

Only a few contributions used hitherto the extended finite element approaches to account for the heterogeneity of the material as performed in [28] and more recently in [29].

The present contribution presents a novel and complementary approach, based on the integration of geometry modelling with discretization using level set descriptions, with the following three original aspects. A RVE generator for woven composites is presented by constructing a level set description of the yarn’s geometry, incorporating geometrical conditions inspired by local equilibrium constraints at the contacts between yarns. This allows enforcing a non-overlapping geometry of the reinforcement, starting from an initial approximate configuration of the yarns, while not imposing a constant cross section of the yarns. This geometrical description allows a seamless transition to subsequent mechanical simulations, based on the eXtended Finite Element Method (XFEM); in which the discretization is independent from the material boundaries that are implicitly taken into account by an enrichment of the displacement field [30].

The paper is organized as follows. The geometrical RVE generator making use of level set functions is first described in Section 2, where the main constituents are introduced in a progressive manner, starting from the main principles and giving details for optimizing the implementation. Section 3 briefly summarizes the principles of the extended finite element framework used in the mechanical simulations, while Section 4 illustrates the use of the weave generator to produce various reinforcement schemes. In Section 5 the use of the integrated frame work is illustrated with the extraction of the in-plane mechanical properties of a 3D reinforced composite. In Section 6 the results obtained are discussed and perspectives detailed of the proposed approach and future work, while conclusions are provided in Section 7.

## 2. A RVE Generator for 3D Reinforced Woven Composites

### 2.1. Principles of the Weave Generator

The objective is to develop a flexible tool allowing the use of XFEM methodology to easily compare the mechanical behavior of different types of reinforced composite systems. In addition to the choice of a weaving scheme, several parameters may affect the reinforcement morphology; among which the tension applied on the yarns, the yarns cross section size and shape, the bending and torsional stiffness of the yarns, as well as the (transversal) stiffness of their cross section and the possible friction between the contacting yarns. The weave generator presented is mainly geometry-based and takes into account in a simplified and implicit manner the tension applied on the yarns, as well as their cross section area, initially assuming circular cross sections for simplicity. This assumption could however be avoided in future developments. The yarns are assumed infinitely compliant with respect to bending, and torsion is not considered. No friction is included in the generation process at this stage, and the transverse stiffness is assumed infinite during the generation process (this assumption is not made for the mechanical simulation part). A non circular cross-section can however be obtained by post-processing at the end of the generation process, as will be explained later. With these simplifying assumptions, a weave is in equilibrium if at each point of each yarn, the resultant of the tension and potential contact forces (with other yarns) vanish. This condition will however not be used explicitly here, and a geometrical condition will be used at the contacting points between yarns instead. Based on a given initial (discretized) approximate configuration of the weaving scheme described by vertices located on the centerline of yarns, the generator will update iteratively the position of vertices until an equilibrated configuration is obtained, *i.e.*, when a stationary position of the vertices is reached.

**Figure 1:**
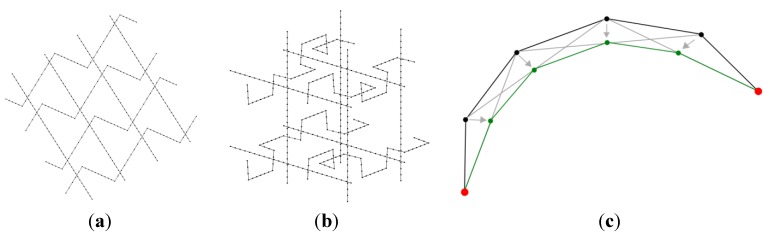
(**a**) Input data used for the weave generation process of a simple weft-warp yarn system; (**b**) 3D weaving similar to that reported in [22]; and (**c**) Fundamental operation (A) for tensioning of the yarns. Black lines represent the position of the yarn centerline prior to the operation (A), while green lines represent its position after operation (A). Grey lines are segments linking the neighbors of each of the vertices. Red dots represent fixed vertices at the extremities of the yarn.

The initial configuration provided for the generator consists of vertices and edges discretizing simple curves (straight segments in the present manuscript) representing the centerlines of the yarns (Figure 1a,b). This data defines the weaving scheme and is the input required from the analyst. All the subsequent operations in the weave geometry determination are independent of the analyst:

1. An iterative procedure updates the initial situation as explained below in Section 2.2 to mimic the yarns behavior under tension based on geometrical arguments. At the contacting points, the relative tension of the contacting yarns at equilibrium is implicitly controlled by the relative displacements of the contacting points to be imposed in order to enforce a non-overlapping configuration.
2. A post-processing of the obtained configuration is used subsequently in order to avoid potential “residual” overlaps or to produce qualitatively and geometrically (*i.e.*, without account of the yarn’s transversal stiffness) noncircular yarn cross sections at contacting regions.

### 2.2. Iterative Procedure

Within the iterative procedure, the following scheme is used to represent geometrically the effect of tension in the yarns. A tensioned yarn without any contact is straight. At each iteration of the iterative process, each vertex of the centerlines of yarns is moved at the center of the segment that links its two neighboring vertices, as depicted in Figure 1c. This operation is denoted operation (A) in the sequel.

**Figure 2:**
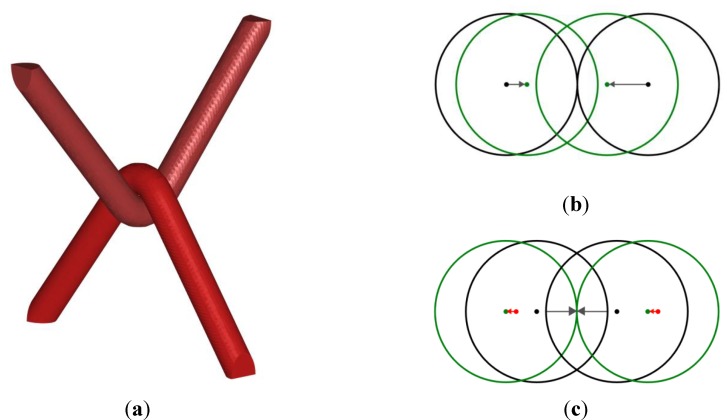
(**a**) Geometry of two equally tensioned contacting yarns; (**b**) and (**c**) schematic representation of fundamental operations (A) and (B) treating geometrical contact between two yarns during one iteration and illustration of the gradual movement of the contact point in the non-equilibrium configuration; (**b**) represents the operation (A); and (**c**) the operation (B). Both yarns are represented as circles for simplicity even if the real 3D geometry is more complex. Black circles depict the initial position, while green color denotes the final position associated with both operations. Red arrows highlight the resulting displacement after the iteration.

When two yarns are contacting, they prevent each other from reaching a single straight shape. With the adopted assumptions, two contacting yarns will form straight segments separated by a kink. If contacting yarns are equally tensioned, each one of them has an equal angle between its two segments; and the vertex forming the angle of one yarn is located on the bisector of the angle of the other yarn, as depicted in Figure 2a. Based on these features, an additional operation (B) can be added next to (A) in each iteration to treat contacting yarns, which consists of the following. First, contact points have to be located, by searching for interpenetrations caused by operation (A) during iterations. These contact points are detected in an automated manner at the vertex level. For each yarn processed, the distance to every other yarn is evaluated and compared with the radius of the processed yarn. This distance is measured as the distance to a poly-line (vertices linked by straight segments) minus a constant representing the radius of the yarn. If the discretization is fine enough, each contact is detected by both involved yarns, both having enough information to process the contact independently. When an interpenetration is detected, each vertex is moved away from the contact in the direction normal to the closest point of the surface with a displacement equal to half of the computed interpenetration, see Figure 2b,c. This procedure enables recovering at the end of the iteration a situation in which the contact is exactly determined. The direction of the vertices displacements is determined by the gradient of the distance to the closest yarn, computed as a by-product during the interpenetration detection. With both operations (A) and (B) repeated in a loop, the situation would become stationary if the displacement produced by both operations was exactly balanced. The magnitude of vertices displacement resulting from operation (A) is proportional to both the angle formed by the segments neighboring the vertices and their lengths. At the contact zones between two yarns, vertices of both yarns are constrained on two curves of the same curvature (equal to 1/(R1+R2) for the case of perpendicular yarns) by operation (B), and the segment lengths remain the only parameters influencing the displacements imposed by operation (A) in these zones. In this case, two yarns with the same initial length and discretization will eventually reach a tensioned configuration minimizing similarly their lengths as in real situations, and will form two equal kink angles. Conversely, if two contacting yarns do not have the same total length (e.g., because the initial situation is made of straight lines and poly-lines of dissimilar lengths with the same number of vertices, as in Figure 1a), operation (A) moves the vertices of the longest yarn more than the other. Then, with operation (B) always moving both yarns by the same amount, the contact point will move from iteration to iteration to reach an equilibrated situation, with both yarns having the same length (Figure 2b,c). As the interpenetration correction is performed in the direction normal to the contacting surfaces, the configuration will only become stable from one iteration to the next when the displacements produced by operation (A) are also normal (*i.e.*, when the centerlines of both yarns intersect the bisector of the angle of the other yarn). 

It is emphasized that this procedure to take account of the yarn tension remains implicit and approximate. As the discretization influences the global numerical process, it is not straightforward to adjust it precisely to obtain realistic tension values. Moreover, the initial yarns’ lengths may be constrained by practical aspects linked to the definition of the weaving scheme by the analyst. Note also that specific situations may arise when more than two yarns are in contact at the same vertex. In this case, the displacements imposed by operation (B) (and deduced from a 2-yarn configuration) may leave the middle yarn’s position unchanged, as it is moved in opposite directions due to the operations (B) applied to both contacts. This leads to residual contacts (of half the interpenetrations); and can be solved (asymptotically) by repeating operation (B) several times for these contacts in the same iteration, and by using the post-processing step (see Section 2.5).

### 2.3. Accounting Implicitly for Different Tensions

**Figure 3:**
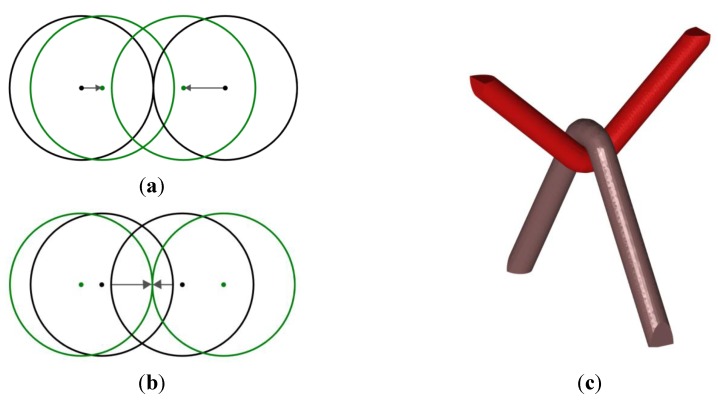
(**a**,**b**) Schematic representation of operation (A) and (B) respectively, with one yarn twice as much affected by operation (B) as the other, the situation is stationary since operation (A) also produces displacements two time larger for this yarn; and (**c**) corresponding configuration obtained for two yarns of the same initial length.

In addition to being approximate, the tensioning procedure presented so far does not allow for taking into account the large tension differences required to obtain quasi-straight weft yarns. This motivates the introduction of an additional feature that allows the operation (B) to move two contacting yarns asymmetrically to reach different tension levels in the contacting yarns. Attributing a different fraction *p* and 1 − *p* of the interpenetration distance to each yarn as a correction displacement in operation (B) allows the process to reach stationary situations reproducing qualitatively real configurations with yarns subjected to different tensions (Figure 3). In particular *p* = 0 or *p* = 1 leads one of the yarns to be exactly straight. Note that the case presented in Section 2.2 matches a value *p* = 0.5. Such a parameter is therefore required as additional data for each couple of types of yarns, and allows taking into account limiting cases of non-tensioned and infinitely tensioned (*i.e.*, straight) yarns. Note that this parameter *p* remains a phenomenological parameter to be chosen *a priori*. The explicit relation between *p* and the related tensions is not investigated here and will be the topic of further refinements of the framework. 

### 2.4. Optimization, Conditions at the Boundaries and Practical Implementation

The global scheme of the iterative procedure presented above needs some additional features to reach a practical implementation.

To ease the definition of the initial configuration, the iterative procedure should be robust enough to be able to start with an initial situation containing overlapping yarns. Describing a weaving scheme with simple sets of segments is quite straightforward, but preventing yarns from overlapping for a given radius requires more advanced curves and becomes cumbersome for complex weaving schemes. The iterative procedure therefore starts with a very small value for the yarn radius and increases it progressively until the actual target value. During this initialization stage, operation (A) is deactivated, and vertices are moved only to prevent overlap by operation (B). When the yarns’ radii reach their actual values and overlaps are resolved at a given tolerance, operation (A) is reactivated and the iterative procedure is executed in the standard way described above.

**Figure 4:**
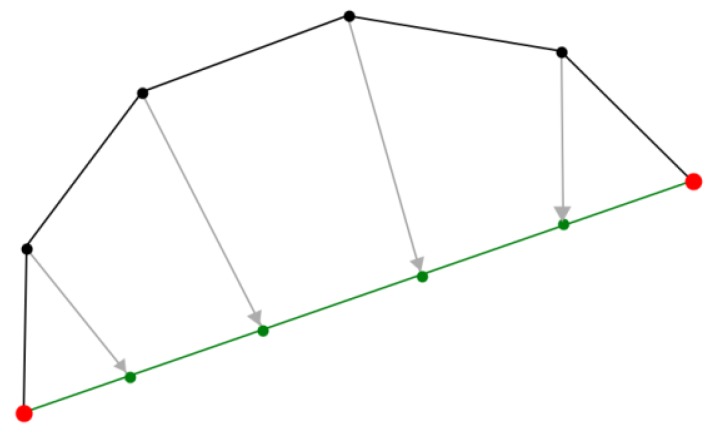
Alignment of the yarns’ centerline description points (vertices) between contacting points to increase the efficiency of the iterative process (red dots in this figure denote two successive contact points along a yarn).

The operation (A) defined in Section 2.2 indeed accounts for yarn tensioning, but the corresponding iterative process is lengthy as a fully tensioned configuration is only obtained asymptotically. Between two contact points, a given yarn should be straight if no contact point remains to be detected. When all contact points are detected in the iterative procedure, this information could be used to execute operation (A) in a more efficient way as follows. The vertices of the yarn between two contact points can be aligned between the contact points in a uniform manner, see Figure 4. This of course relies on the availability of a good approximation of the contact configuration for the yarns, and this improvement can only be used when such an approximation is reached. Practically, this procedure is only used when the required displacements of the vertices are smaller than a given value, taken as a fraction of the yarns’ radii. This distance limitation prevents yarns from passing through each other during operation (A) and ensures that all contacts are detected and will be brought back by operation (B).

Conditions have to be defined for the displacements imposed during the generation process at the boundary of the considered RVE. The simplest solution consists in preventing any displacement of the yarn vertices located on the RVE boundaries. However, the computational homogenization procedures usually make use of periodic boundary conditions [31], especially suited here as the textile produced is assumed periodic. Practically, periodicity of the reinforcement is achieved here by enforcing the following constraints:

- Vertices located on the RVE boundary are constrained to remain on that planar boundary (planar constraint);
- Yarns are looped at opposite boundaries of the RVE, *i.e.*, the first and last vertices of a yarn will use the one but last and second vertices respectively to perform operation (A);
- Finally, for the detection of contacts and for the post-processing step (see Section 2.5), distances of points with respect to the yarns’ surfaces have to be computed in a periodic manner.

Finally; it can be emphasized that if initial circular cross sections of yarns are assumed here for simplicity; the concepts described so far can be extended to other cross section shapes; such as lenticular (elliptical) cross sections. In particular; the level set formalism allows an easy extension to such other shapes; which could be more cumbersome to accommodate using the approach of [22] based on inflatable tubes.

### 2.5. Post-Processing for Residual Contacts

After the iterative procedure involving operations (A) and (B), some residual interpenetrations may still be present in spite of operation (B), for instance when more than two yarns are overlapping at the same position. Such interpenetration has to be suppressed to ensure a proper execution of the mechanical simulation in the sequel. This is achieved using an implicit representation of the yarns’ geometry through level set functions based on distance fields. This type of approach was proposed in [32], and consists in starting from distance fields from several objects (yarns in this case), to recombine them to define new functions, and to use the latter ones to extract new geometries defined as based on their iso-level surfaces.

Let us denote *DS_i_*(*x*) the distance field to a given yarn *i*, and *DS*_0_(*x*) the distance field to all the yarns present except yarn *i*. *DS*_0_(*x*) can be computed as

(1) $$DS_{0}\left(x\right)=min\left(DS_{j}\left(x\right)\right)\;\;\;\;\;\;with\;j\neq i$$

**Figure 5:**
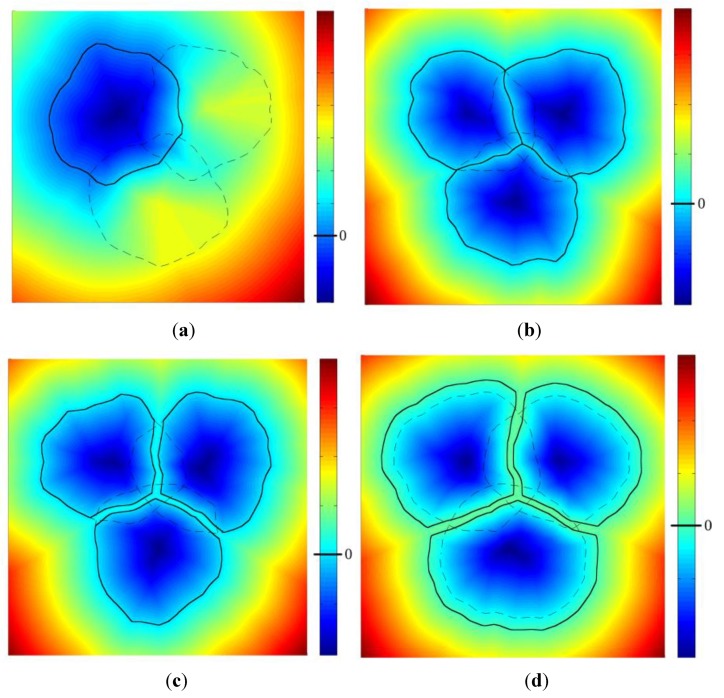
(**a**) 2D representation of the function *DS_i_*(*x*) − *DS*_0_(*x*); (**b**) use of level set functions to eliminate residual interpenetration zones on arbitrary shapes, dashed lines are original shapes and full lines are the final situation. The background function is min*_i_*$\left(O_{i}\left(x\right)\right)$; (**c**) introduction of a clearance between yarns for further processing by eXtended Finite Element Method (XFEM) mechanical simulations; and (**d**) increase of the yarns’ diameters to mimic contact zone deformations of the yarns’ cross section.

The function *DS_i_*(*x*) − *DS*_0_(*x*) takes negative values in any point closer to yarn *i* than to the other yarns, and positive values elsewhere. If yarn *i* does not overlap with any other yarn, the zero level surface of this function is a closed surface that encloses the yarn *i*, and that encloses all the points that are closer to yarn *i* than to the others, see Figure 5a for a 2D illustration. Conversely, if yarn *i* does overlap another yarn, this closed surface cuts the interpenetration zone in two parts, being locally the surface of equal distance between the boundaries of both yarns. The intersection between the volume bounded by the zero-level surface of the function *DS_i_*(*x*) − *DS*_0_(*x*) and the original yarn *i* defined by

(2) $$Q_{i}(x)\;=\;\text{max}(DS_{i}\left(x\right),\;\;\;DS_{\text{j}}\left(x\right)\;-DS_{0}\left(x\right))$$

matches the original shape of yarn *i* from which half of the interpenetration zone is subtracted (in this relation *i* denotes the yarn *i*). This surface can be extracted by contouring *O_i_*(*x*) at its zero level. If this operation is performed for all the yarns, the overlapping zone can be transformed into an exact contact, modifying locally the shape of the cross section of yarns (Figure 5b). Subtracting a constant term from the function *O_i_*(*x*) defined in relationship (2) before taking its zero-level surface furthermore allows introduction of a (uniform) clearance as described in Figure 5c.

Note that this post-processing step may be used also if one wishes to obtain non circular shapes for the yarns of the textile reinforcement due to contacts. This can be achieved by artificially increasing the yarns’ diameters with respect to their real values, thereby causing important interpenetrations, which are then treated similarly with relationship (2), see Figure 5d. It is however emphasized that this corresponds to a geometrical treatment and by no means results from the actual transversal deformability of the yarns at this stage of implementation. It is also noted that the level set functions introduced by relationship (2) can be used to construct the enrichment of the displacement field within an extended finite element approach as briefly explained in Section 3.

## 3. EXtended Finite Element Method (XFEM) and Computational Homogenization

The difficulties in generating finite element meshes for complex microstructures triggered the development of alternative discretization strategies. The eXtended Finite Element Method (XFEM) for heterogeneous materials was developed by Moës and co-workers [28] for this purpose, but was rarely used for woven composites, see [29]. Its essential feature is that it does not require meshes to conform to the material boundaries inside the microstructure, these being accommodated by additional terms in the discretized fields. The method is based on the Partition of Unity principle and makes use of level set functions to describe the RVE internal material boundaries and the related enrichment of the displacement field. This allows its seamless integration with the RVE generator.

**Figure 6:**
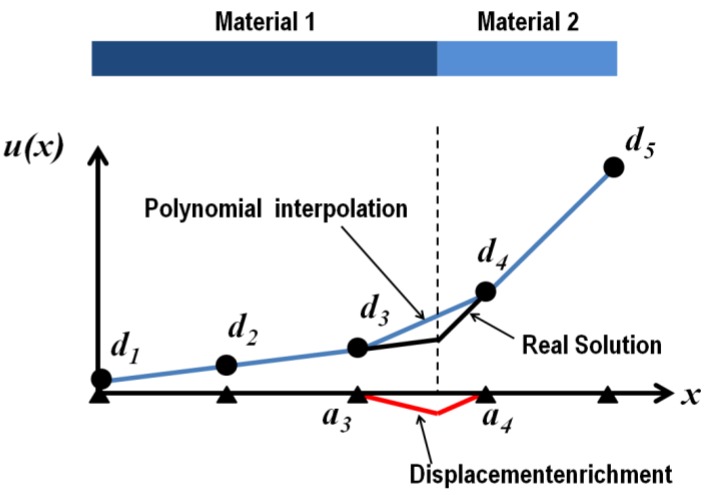
Principle of eXtended Finite Element enrichment by a level set function for a bi-material 1D example. The polynomial variation described by nodal displacements (blue curve with related *d* unknowns) is complemented by a nodal-based enrichment with limited support (red curve with *a* unknowns) to obtain the real displacement field without placing nodes at the material interface.

The principle of XFEM is to use a non-conforming regular mesh with additional degrees of freedom related to additional shape functions (denoted the enrichment), introducing strain jumps induced by material heterogeneities. This treatment, concentrating on finite elements intersected by a material interface (e.g., inclusion/matrix boundary), uses level set functions to construct the enrichment and to subdivide elements by material at the stiffness integration stage. The interpolation of each displacement field component inside a given finite element therefore reads

(3) $$u\left(x\right)=\underset{i}{\sum }N_{i}\left(x\right)d_{i}+\underset{j}{\sum }N_{j}\left(x\right)\text{ Ψ}\left(x\right)a_{j}$$

where the index *i* now refers to the nodes of a given finite element; *d_i_* being the regular nodal displacements. The first summation represents the usual finite element polynomial interpolation containing the standard shape functions as a partition of unity. The second sum introduces the enrichment with *a_j_* the additional unknowns and Ψ(*x*) the enrichment functions. For heterogeneous materials, the level set function was shown to be a good basis to introduce the required strain jump at the material boundary. This principle is illustrated in Figure 6 for the simple case of a 1D structure. In the present contribution, the function defined by relationship (2) was used in order to construct the enrichment Ψ(*x*) based on nodal values of the level set function (index *j* refers here to nodes) according to

(4) $$\Psi \left(x\right)=\underset{j}{\sum }\left(N_{j}\left(x\right)\left|O_{j}\right|-\left|N_{j}\left(x\right)O_{j}\right|\right)$$

The stiffness matrix of the elements intersected by discontinuities has to be integrated separately, attributing each of the material properties to the corresponding domains. Nodal level set values are used to construct the intersections with the implicit interface in a consistent way with the enrichment. These intersections are then used together with element nodes to build sub-tetrahedra completely located in single material domains. A Gauss integration is performed in these sub-tetrahedra attributing material properties to integration points according to the sign of the level set function values, which are interpolated from nodal values.

**Figure 7:**
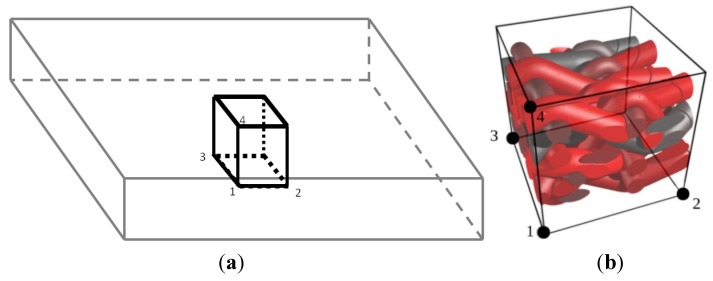
Representative volume element (RVE) controlling nodes using periodic boundary conditions. (**a**) RVE within a plate/shell structure; and (**b**) macroscopic loading control points of the RVE.

The XFEM methodology is coupled with computational homogenization using periodic boundary conditions. The averaged mechanical properties of a heterogeneous material can be deduced by loading a RVE containing the main fine scale features of the material, and solving the corresponding equilibrium problem. When a macroscopic strain ***E*** is applied to a RVE, the displacement of a point inside the RVE is given by

(5) $$\vec{u}\left(\vec{x}\right)=E\cdot \vec{x}+{\vec{u}}_{f}\left(\vec{x}\right)$$

in which $\vec{x}$ is the position of the point within the RVE and ${\vec{u}}_{f}$ is a fluctuation associated with the heterogeneity of the material. Assuming a periodic fluctuation, the average of the fine-scale strain field **ε** resulting from (5) can be shown to be equal to ***E***. Next, the Hill-Mandel condition (energy equivalence between the fine-scale and macroscopic descriptions)

(6) $$\Sigma :\delta E=\frac{1}{V}\int_{V}\sigma :\delta \varepsilon \;dV$$

implies that the macroscopic stress tensor **Σ** is obtained as the volume average of the microstructural stress tensor **σ**. Using periodicity, the macroscopic stress tensor is obtained based on the RVE tying forces at nodes controlling the macroscopic loading as

(7) $$\Sigma \;=\;\frac{1}{V}\int_{V}\sigma \;\text{d}V=\frac{1}{V}\overset{4}{\underset{a=1}{\sum }}{\vec{f}}^{\left(a\right)}{\vec{x}}^{\left(a\right)}$$

where the summation spans the nodes controlling the RVE loading [31]. The periodicity of the fluctuation is enforced by linear connections between corresponding faces. Four controlling points (denoted 1 to 4 in Figure 7) are used to apply the macroscopic stress or deformation modes of the RVE, provided identical meshes are used on the opposite faces of the RVE. In the present case, illustrations will be given for RVEs periodic in all directions, as well as for RVEs representing the in-plane behavior of in-plane loaded plates (with top and bottom faces remaining traction free). In this latter case, only in-plane loading of the RVE needs to be considered; and in-plane periodicity only is assumed, while the periodicity condition is relaxed along the thickness direction to model the behavior with unconstrained top and bottom faces.

## 4. Weave and RVE Generation Illustrations 

The weave generation tool is now exploited to illustrate its versatility.

**Figure 8:**
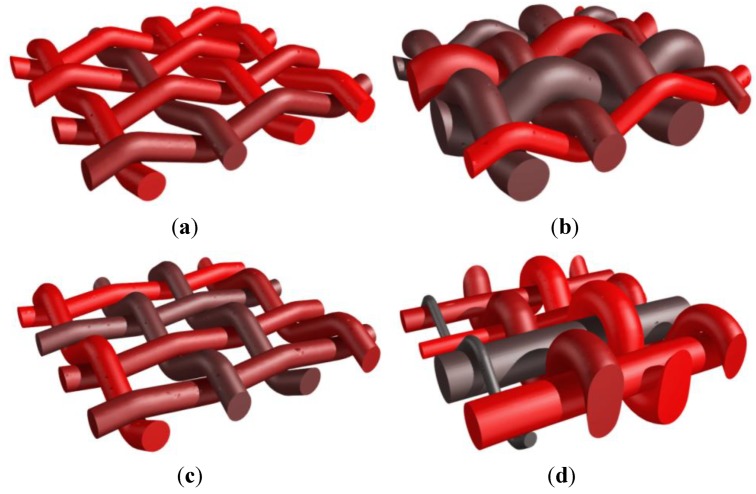
Generation of a reinforcement scheme for a one layer thick weaving. (**a**) Single set of yarns in orthogonal directions with same diameters; (**b**) same weaving scheme as (**a**) with different diameters of yarns; (**c**) same weaving scheme with increase in the tension of one family of yarns; and (**d**) combination of diameter change with fully tensioned yarns.

A simple weaving of two sets of yarns is first used to illustrate the effects of changing the geometry and the tension of the yarns. A single ply of yarns is considered in both directions. Figure 8a illustrates the shape obtained when an equal amount of tension is considered for both families of yarns [*p* = 0.5 in operation (B)] for both orientations. The same example is also treated using different diameters for one of the plies of yarns (one orientation) on Figure 8b. In this second case, the contact conditions implemented in operation (B) take into account this geometry change. The effect of a modification of the tension level in the yarns in one orientation is depicted in Figure 8c, with a factor *p*_weft_ = 0.3. The weft yarns undergoing a higher tension exhibit a straighter shape. This effect is even more pronounced in Figure 8d where an almost completely straight family of yarns is obtained, with *p*_weft_ = 0.05. In this case, this tension difference is combined with different diameters for the yarns to show the robustness of the weave geometry generation.

**Figure 9:**
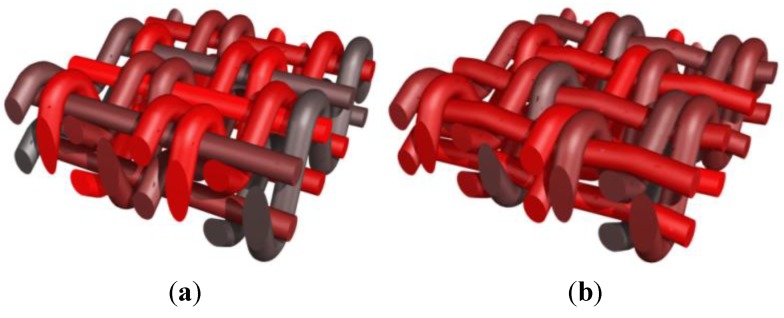

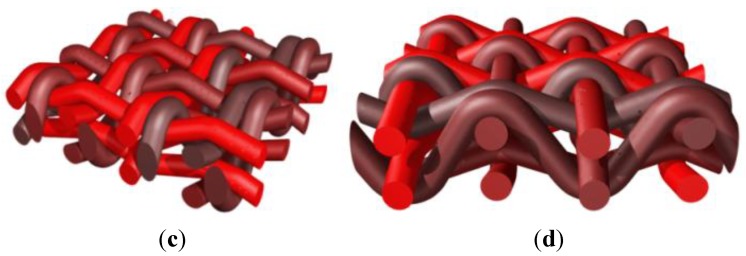
Generation of a reinforcement scheme with two families of warp yarns alternating on respectively one and two sets of weft yarns; (**a**) fully tensioned weft yarns; (**b**) effect of a decrease in weft yarns tension; (**c**) further decrease of the weft yarns—axonometric view; and (**d**) further decrease of the weft yarns—side view.

A second series of more complex weaves is performed next to illustrate the possibility to reach important volume fractions of reinforcing textile. In this case, two plies of weft yarns are used that are fully tensioned with respect to the orthogonal (warp) yarns. In the orthogonal direction two different vertical planes are considered within a period of the reinforcement arrangement. In a first plane two warp yarns oscillate around one level of weft yarns. In a second vertical plane, one single warp yarn oscillates across both plies of weft yarns (binders). A first version is presented with weft yarns completely tensioned in Figure 9a. When the conditions on relative angles in yarn contacts [in operation (B), with parameter *p*] are relaxed, the weft yarns are slightly deformed as shown on Figure 9b. When the condition on the angles is identical at the contact between both orthogonal directions (*i.e.*, *p* = 0.5 for all contacts between yarns), the presence of two different types of warp yarns (the ones oscillating around each weft and the binders) implies different angles of contact with the weft yarns as depicted in Figure 9c,d. Furthermore, the binders and the warp yarns are in this case in contact with each other as a result of the undulations of the “weft” yarns.

**Figure 10:**
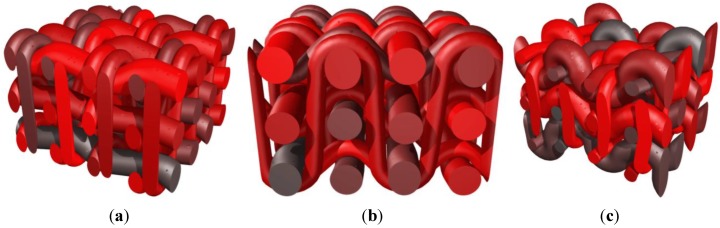
Reinforcement scheme with warp yarns and binders alternating on three plies of weft yarns. (**a**) Normally tensioned weft yarns (*p*_weft_ = 0.3)—axonometric view; (**b**) normally tensioned weft yarns—side view; and (**c**) effect of excessive tension in binders (*p*_weft_ = 0.8).

A similar pattern is reproduced in Figure 10a,b but for binders spanning now three plies of warp yarns. As a result of the angle condition (tension) on the contacts, the weft yarns are undulated in different ways. Both outer plies of weft yarns are more oscillating as opposed to the central ply, because of the presence of the warp yarns and the binders at these places. As can be seen in Figure 10a,b, the periodicity conditions are correctly taken into account in the definition of the weave (this is particularly obvious for the binders). Note also that when the binders are excessively tensioned, the weave pattern becomes much more complex (Figure 10c).

**Figure 11:**
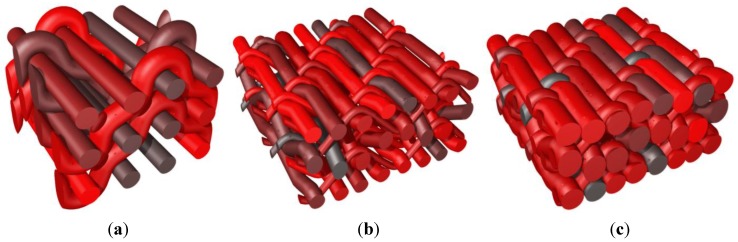
Woven composite with post-processing of the weave configuration to reach contact between yarns. (**a**) Weaving principle; (**b**) RVE before level set-based post-processing; and (**c**) RVE after level set-based post-processing.

To illustrate the potential effect of post-processing, a woven composite is now considered as in Figure 11a. By adapting the diameters of the yarns, a more compact form can be obtained, as depicted in Figure 11b. This configuration is then obtained as input for the post-processing step that consists in increasing the cross section of the yarns as illustrated in Figure 11c.

**Figure 12:**
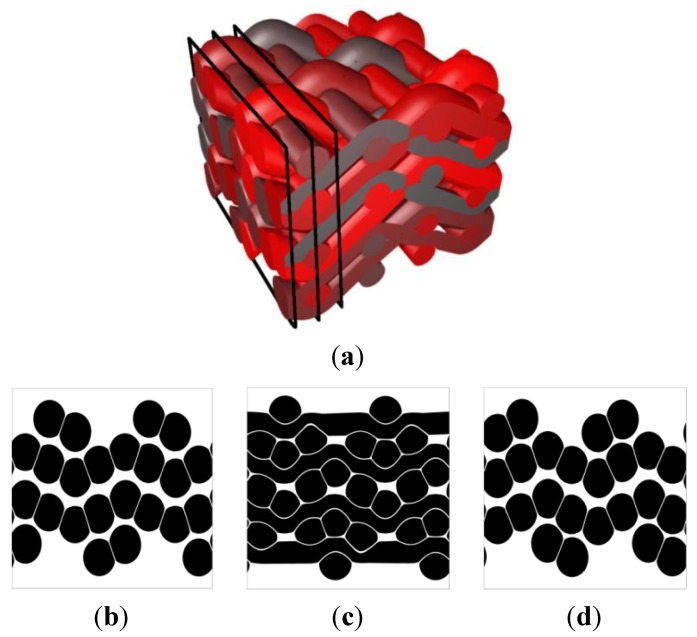
Woven composite with post-processing of the weave configuration to reach contact between yarns; (**a**) axonometric view; and (**b**–**d**) cuts of the weave perpendicular to the warp yarns.

This can be further illustrated by the texture represented in Figure 12. A woven composite is obtained using the level set-based post-processing tool to enlarge the yarn cross sections. This post-processing tool can subsequently be used to modify the shape of the yarns at the contacting surfaces. A small clearance is added at the contacting surfaces as depicted in Figure 12b–d. Note however that this shape modification is purely geometrical and does not incorporate the real (transversal) mechanical properties of the yarns.

**Figure 13:**
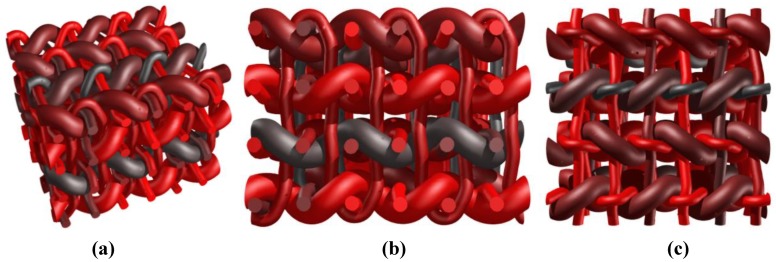
Generation of a 3D reinforced composite similar to that studied in [22]. (**a**) Axonometric view; (**b**) side view illustrating binders in thickness direction; and (**c**) top view.

Finally a RVE of a weaving scheme similar to that presented in [22] is given in Figure 13, corresponding to a 3D reinforcement. Three types of yarns are considered: warp yarns, horizontal weft yarns, as well as vertical binders looping in the depth around the horizontal weft yarns.

## 5. Homogenization of Woven and 3D Composites

A simple parametric study is now given to illustrate the use of the presented framework, by homogenizing the tensile elastic behavior of woven composites. The variation of both the morphological and material parameters is investigated, as well as the RVE boundary conditions used for the homogenization procedure. Three different RVEs and five material parameter sets are used to obtain a simple but illustrative set of results. 

The RVEs are assumed to represent the average behavior of a planar structure (plate) subjected to in-plane loading. They are defined from the weaving scheme used in [22] which consists of a bound stacking of four layers of warp and horizontal weft yarns. In addition to alternate up and down around horizontal weft yarns, warp yarns also alternate right and left around vertically oriented binders, that can thus be considered as vertical weft yarns when ignoring the outer layers in which binders are actually looped, as illustrated in Figure 13. In [22], the two inner layers of the weaving were studied, assuming binders as purely vertical (continuous) weft yarns and applying full 3D periodicity conditions for the homogenization procedure. This resulted in the consideration of an “internal” RVE in [22], without accounting for the outer layers. These outer layers with looped binders must however be accounted for in the overall behavior of the composite, especially if plate structures are considered. In such cases, restricted RVE periodicity conditions should be used for proper representation, letting the top and bottom faces of the plate through thickness RVE traction free, while keeping in-plane periodicity constraints on other boundaries.

The three RVEs with their boundary conditions—full periodicity in all directions and in-plane periodicity only with traction free top and bottom faces—are now compared. Two RVEs are 4-layer weavings, including outer layers and binder loops; while the third is also a 4-layer weaving but in which all the layers are considered to be inner layers. Such an approximation, sometimes used to simplify the microstructure, consists of assuming that the binder loops are not included, as if this RVE was the inner part of a 6-layer weaving. In this last case, the binders are therefore replaced by vertical wefts.

**Figure 14:**
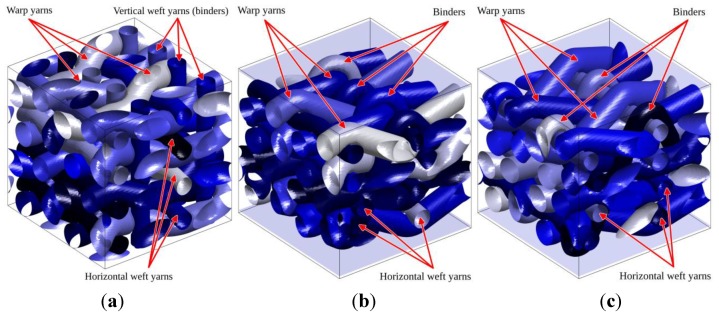
RVEs investigated using the level set-based XFEM simulations: (**a**) 3D RVE with vertical wefts; (**b**) 2.5Da RVE with non-alternating looped binders; and (**c**) 2.5Db RVE with alternating looped binders**.**

These RVEs are denoted respectively 2.5D and 3D RVEs. The 3D RVE is homogenized with full periodicity conditions (thus applying periodicity conditions on faces perpendicular to vertical wefts), while for the two 2.5D RVEs traction free top and bottom faces are considered. It is emphasized that 2.5D RVEs exhibit a clearly different internal initial morphology compared to the 3D one due to the tension in (looped) binders that has the effect of compacting the inner layers and displace yarns. This effect is obviously not present in the 3D RVE as vertical wefts are not looped and tend to be straight, as their tension is increased as illustrated in Figure 14a. Both 2.5D RVEs slightly differ through their binder position as illustrated in Figure 14 and Figure 16e–f. In the weaving illustrated in [22], all binders are looped around the same horizontal weft yarn. This causes the top and bottom horizontal weft yarns to reach very different configurations, the binder looping side being more compacted than the opposite one. By alternating the weft yarn around which binders loop, a more regular configuration can be obtained, for which either a single warp yarn or binder pulls inward an outer layer of weft yarn; instead of a configuration in which either both the warp yarn and binders act together, or none of them is present. To compare those two situations, both 2.5D RVEs present either non-alternating binders (2.5Da) or alternating binders (2.5Db), as illustrated in Figure 14b,c (see also Figure 16e,f for an isolated view of the binders in both 2.5D RVEs).

Obtaining actual material parameters for the constituents can be difficult, especially for the anisotropic properties of yarns, which depend on their fibrous microstructure and on their degree of impregnation. The transverse yarn properties are therefore usually approximated by homogenization schemes based on their microstructure, as discussed in [25,33]. A set of anisotropic parametersis therefore used here, covering a wide range of behavior from full isotropy to a ~1/5 transverse anisotropy. The isotropic situation is built on longitudinal properties of yarns taken from [22], and a transversely anisotropic behavior of the yarns is obtained by decreasing transversal parameters (both Young and shear moduli). This allows investigating the impact of a variation of these uncertain parameters both on the homogenized behavior and on the local (microstructural) stress fields obtained. The material parameter sets used for the computations are given in Table 1.

**Table 1:** Material properties of the constituents. The yarn properties are mentioned.

Set	1	2	3	4	5
E_m_ (GPa) (Matrix)	3				
v_m_ (−) (Matrix)	0.35				
E_l_ (GPa) (Yarns)	230	230	230	230	230
E_t_ (GPa) (Yarns)	230	200	150	100	50
v_tt_ (−) (Yarns)	0.225	0.2285	0.2343	0.2400	0.2458
v_lt_ (−) (Yarns)	0.225	0.2215	0.2157	0.2099	0.2042
G_lt_ (GPa) (Yarns)	93.878	71.745	42.176	21.870	10.960

The RVEs used in the computations are generated with the procedure presented in Section 2 and are assumed of cubic shape. The binder and horizontal weft yarns have a diameter of 6% of the RVE size, while the warp yarns have a diameter of 8% of the RVE size. In the contacting areas between yarns, a clearance of 1% of the RVE size is used in the level-set based post-processing.

The summary of the computations performed is as follows: for each RVE, a tensile test is performed using the five parameter sets. The loading consists of a uniaxial macroscopic tension corresponding to a 1% tensile strain in the warp yarn direction (x). No macroscopic tension or constraint is applied in the *y* and *z* directions. The RVEs 2.5Da and 2.5Db are homogenized with periodic kinematic constraints on *x* and *y* direction only, while the RVE 3D is fully periodic.

**Figure 15:**
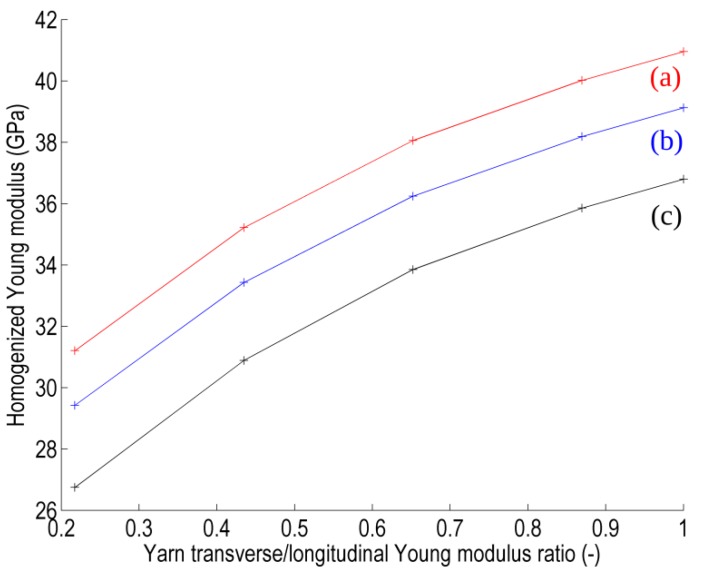
Homogenized Young modulus of RVEs (**a**) 2.5Da RVE with non-alternating binders; (**b**) 2.5Db RVE with alternating binders; and (**c**) fully 3D RVE with purely vertical (weft) binders.

First, a general overview is given in Figure 15 depicting the homogenized Young modulus evolution as a function of the yarns anisotropy ratio (E*_t_*/E_l_) for each RVE. All three RVEs exhibit the same trend but with substantially different values. In particular, the 3D RVE yields a lower stiffness of the composite with respect to the 2.5D RVEs which are believed to be better representations of a real planar (plate) composite structure. The lower stiffness obtained with the 3D RVE is also potentially accompanied by stress underestimation with respect to the 2.5D RVEs. In addition, the difference between the two 2.5D RVEs resulting from the binders position difference is clearly visible, indicating that the alternate RVE (2.5Db) is softer than the other (2.5Da) and potentially that lower peak stresses can be expected.

Excessive interfacial tangential stress is one possible reason for decohesion between the yarn and the matrix material, which in turn has a major influence on the composite life time and service integrity. With **σ** being the stress tensor and *n* the outward unit normal at the yarns surface, this interfacial tangential stress is given by

(8) $$\sigma_{t}=\;\left|\sigma \cdot \vec{n}-\left(\vec{n}\cdot \sigma \cdot \vec{n}\right)\cdot \vec{n}\right|$$

**Figure 16:**
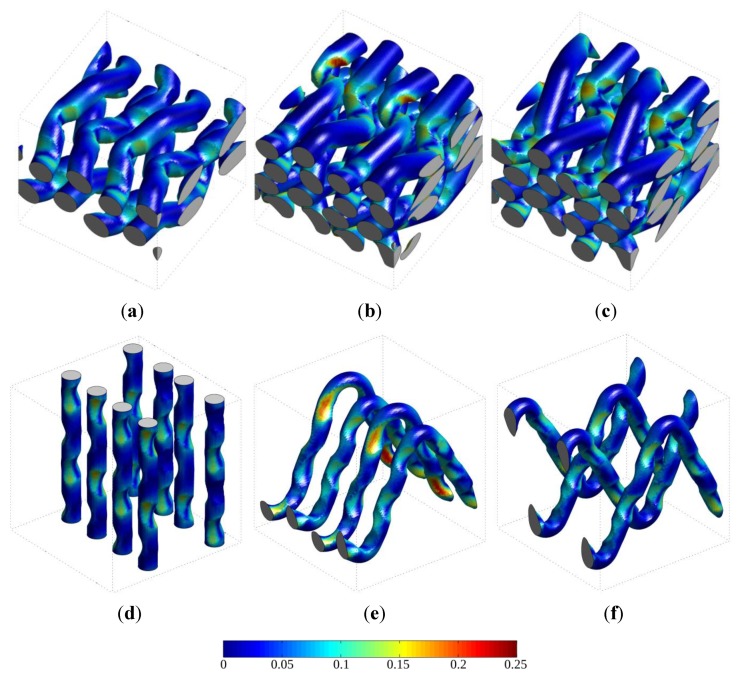
Interfacial tangential stress (relationship (8)) for a tensile strain of 1% in the warp average direction for the three considered RVEs. (**a**–**c**) Interface between warp yarns and matrix; and (**d**–**f**) Interface between binders (2.5D RVEs) or vertical wefts (3D RVEs) and matrix. The maximum stress value in the scale is 0.25 GPa.

The stress levels are illustrated for warp yarns and binders (vertical wefts for the 3D RVE) in Figure 16 for a qualitative comparison of RVEs using the parameter set 5. These figures show that the non-alternating binders configuration (RVE 2.5Da) indeed leads to higher tangential stresses with respect to the alternating configuration (RVE 2.5Db) at the interface between the warp yarns and the matrix (top row of Figure 16), as well as between the binders and the matrix (bottom row of Figure 16).

**Figure 17:**
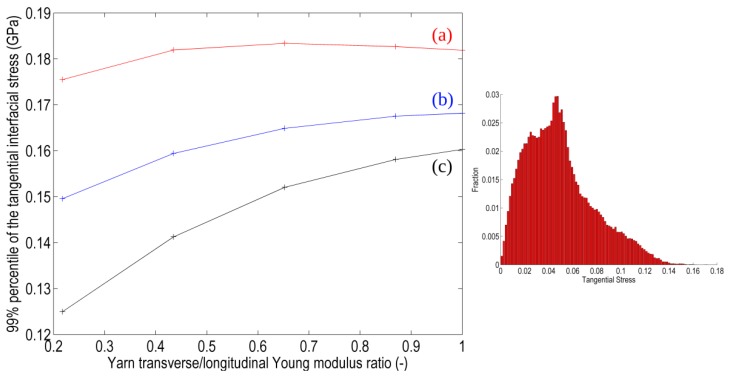
Maximal interfacial tangential stress present in the RVE as a function of the anisotropy ratio in the transversely isotropic behavior of yarns for the three RVEs: (**a**) 2.5Da RVE with non-alternating binders; (**b**) 2.5Db RVE with alternating binders; and (**c**) fully 3D RVE with purely vertical (weft) binders.

A more quantitative comparison is given in Figure 17, depicting the maximal tangential interfacial stress in the RVE for the different RVEs and as a function of the anisotropy ratio of the yarns’ properties. The maximal value of this stress quantity is taken as the 99% percentile of its distribution at the matrix-reinforcement interface and a typical distribution of the tangential interfacial stress obtained in computations is shown on the inset. 

Contrary to the trend observed for the homogenized Young modulus, here the three RVEs do not exhibit the same behavior. The peak tangential interfacial stress remains almost constant for RVE 2.5Da, which suggests that this configuration is less sensible to the yarn behavior than both other RVEs. It also suggests that the assessment of the local stress fields associated with reinforcement with binder using the simplified hypothesis of purely vertical wefts here represented by the 3D RVE can lead to strong approximations.

**Figure 18:**
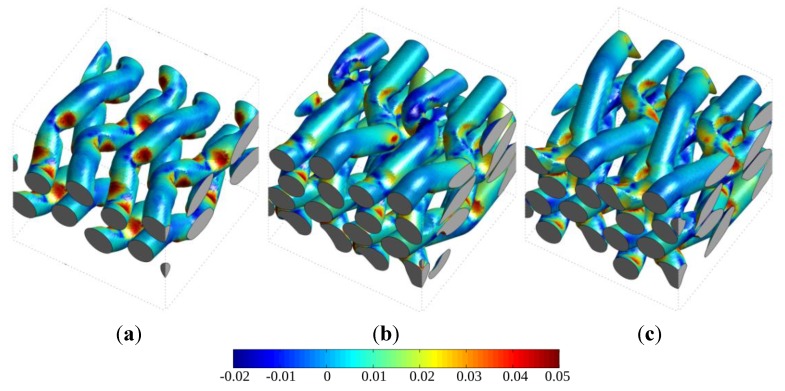
Difference in interfacial tangential stresses between the isotropic (set 1) and transversely isotropic (set 5) assumptions for the behavior of yarns. (**a**) 3D RVE; (**b**) 2.5Da RVE; and (**c**) 2.5Db RVE. The maximum value in the scale is 0.05 GPa.

Finally, the influence of the anisotropy ratio postulated for the yarns on the interfacial stresses can also be depicted using the difference of these stresses obtained locally for set 1 and set 5 of the material parameters, as performed in Figure 18. As expected, accounting for the anisotropy of yarns generally decreases the interfacial stress (positive area). However, stress increases are observed in some locations, in particular on warp yarns close to binder loops. These loci are part of the most loaded part of yarns, particularly noticeable on RVE 2.5Da.

## 6. Discussion and Perspectives

In view of the current development of woven and 3D-reinforced composite systems, computational simulations remain a powerful investigation tool to complement experimental investigations. Computational homogenization is frequently used nowadays to increase the understanding of composite systems, allowing both averaged properties and local field values to be determined. Yet, the complexity of the structure of these material systems renders the modelling task difficult, in particular to build Representative Volume Elements and to discretize them in simulations.

This contribution presented an integrated methodology to model the mechanical behavior of textile-reinforced composites. The reinforcing textile configuration is determined based on geometrical considerations related to the contact between reinforcing yarns, accounting implicitly for the relative level of tension between the contacting yarns. A level set-based post-processing step is incorporated in the generation process in order to prevent any interpenetration of yarns and to represent the “flattening” of the yarns’ cross sections in contacting regions. This allows for a straightforward transition towards XFEM mechanical simulations to determine the average properties of the composite systems. The application of the proposed procedure was illustrated in view of the textile generation process, showing the versatility of the RVE generation procedure. Various reinforcing textile configurations can be generated based on a relatively simple input by the user, including full 3D reinforcements. 

It was shown that the typical configuration studied by [22] can be obtained using the proposed generation process. The extraction of the average mechanical properties as a function of the fine scale properties (geometry of reinforcement, mechanical properties of the yarns, *etc*.) was illustrated in Section 5 by varying the morphology of the reinforcement, as well as the transversal properties of yarns.

It is emphasized that the proposed methodology for RVE generation is complementary to that presented by [22], in which the reinforcing textile geometry is obtained based on inflatable tubes. The latter approach was implemented with a trial to account for the transverse mechanical properties of yarns in the inflatable tubes. While the RVE generation approach presented here does not account for the mechanical properties of these yarns, this aspect will be a topic of future development of the framework. A common limiting assumption of both approaches at this stage consists in the assumed circular (initial) shape of the reinforcing yarns. For the methodology presented in this paper, this assumption could be avoided in future work by adapting the contact detection scheme for more general cross section shapes. For fully general cross sections, this would have an impact on the cost of distance evaluations, as an explicit discretization of the yarn cross section would be required instead of a vertex-based approach. For the case of elliptical cross sections, the shape can be described using a closed-form expression, but an additional “degree of freedom” should be added at the vertex level in order to take into account the rotation of the cross section around the centerline of the yarn. All the other features of the proposed generation process would however remain unchanged, and in particular, the level set-based post-processing treatment.

As opposed to most of the existing approaches, the mesh generation is here made completely independent of the geometry of the matrix and reinforcing phases, as in [29]. A clearance between yarns in which the matrix material is considered is however still required at this stage at the contacting surfaces between yarns in order to facilitate the mechanical simulation. This “added layer” of matrix material is also considered in most of the current approaches in the literature, see [19] for instance. The present XFEM discretization however allows envisioning the replacement of this clearance by a true contact condition between yarns. This can be achieved based on a different displacement field enrichment introducing a potential displacement jump at the interface between yarns, together with a postulated interface material response, as implemented in [34] for concrete cracking. Note also that the same ingredient could be used for modelling debonding between the yarns and the matrix phase.

## 7. Conclusions

The present paper proposed an integrated tool for the modelling of textile-reinforced composites, combining the generation of RVEs with a level-set based extended finite element simulation tool. The RVE generation procedure is based on an iterative scheme to treat contact conditions, and accounts implicitly for the relative tensions in the yarns constituting the reinforcing textile. The interpenetration between the reinforcing yarns is avoided based on a level set approach. This feature subsequently allows an immediate seamless transition towards the extended finite element framework, that allows uncoupling of the discretization from the material boundaries within the RVE and thereby allows simplifying substantially the meshing operations.

The RVE generation was illustrated by means of different types of RVEs of increasing complexity. Starting from simple reinforcing schemes, RVEs containing high volume fractions of reinforcement were generated, including 3D reinforcements and illustrating the flattening effect obtained when introducing a clearance between yarns. Extended finite element simulations were then performed to compare 3D and 2.5D reinforced composites based on their homogenized elastic behavior and the corresponding local stress fields at the interface of the yarns-matrix.

The proposed methodology can be used in future developments to incorporate features of the yarns’ material behavior in the RVE generation procedure, as well as to account for the debonding of the yarns-matrix in the extended finite element mechanical simulations.
